# The Prevalence of Atopic Diseases Among Pediatric Food Allergic Patients

**DOI:** 10.7759/cureus.42149

**Published:** 2023-07-19

**Authors:** Waleed Batais, Bsaim A Altirkistani, Anas M Alswat, Ali A Almehmadi, Waleed A Shaykhayn, Ahmad M Kushari, Loie T Goronfolah

**Affiliations:** 1 College of Medicine, King Saud Bin Abdulaziz University for Health Sciences, Jeddah, SAU; 2 College of Medicine, King Abdullah International Medical Research Center, Jeddah, SAU; 3 Collage of Medicine, King Saud Bin Abdulaziz University for Health Sciences, Jeddah, SAU; 4 Pediatric Immunology, King Abdulaziz Medical City, Ministry of National Guard - Health Affairs, Jeddah, SAU

**Keywords:** pediatric, allergic rhinitis, atopic dermatitis, asthma, food allergies

## Abstract

Introduction

Food allergy is an immunological reaction that occurs in response to particular proteins in food. As food allergy can affect multiple body organs, symptoms usually include pruritus, urticaria, rash, cough, dyspnea, and wheezing. Atopic diseases are common in patients with food allergies, and multiple significant associations have been made between them. As such, the presence of food allergy in atopic patients can be used as an indicator of disease severity. The aim of this study was to estimate the prevalence of atopic diseases in food allergy patients.

Methodology

This was a retrospective cohort study that included pediatric patients aged 0-14 with confirmed food allergies between January 2016 and June 2022. Data was retrieved from electronic medical records and included patients' demographics, type of food allergens, symptoms of food allergy, presence of atopic diseases, and the utilization of healthcare services. Categorical variables were reported as frequencies and percentages, with Chi-squared being used for comparison.

Results

A total of 228 patients were included in the study. Half of them (114, 50%) were diagnosed with an atopic disease. Seventy-four (64.9%) had asthma, 57 (50%) had atopic dermatitis, and 45 (39.5%) had allergic rhinitis. The most common food allergens in atopic patients were eggs and milk products, in 53 (46.4%) and 42 (36.8%), respectively. There was a significant association between allergy to eggs (p=0.0005), bananas (p=0.0242), and strawberries (p=0.0393), and the presence of an atopic disease. No significant difference was found between atopic and non-atopic patients in terms of utilization of outpatient (p=0.09), inpatient (p=0.50), or ER visits (p=0.31) due to food allergy.

Conclusion

The current study demonstrates the prevalence of atopic disease in patients with food allergies. Although certain foods were associated with the prevalence of atopic diseases, both atopic and non-atopic patients have similar utilization of health care services such as outpatient, inpatient, and ER.

## Introduction

Food allergy is defined as an immune response that happens when the body exhibits particular interactions with proteins in the food [1,2]. The immune response could be non-immunoglobulin (IgE)-mediated or immunoglobulin (IgE)-mediated, with the latter requiring prior sensitization to the food allergen and production of symptoms upon re-exposure to the same allergen [2]. Theoretically, any food can cause allergies, but the majority of allergies are caused by certain foods, such as eggs, milk, peanuts, wheat, shellfish, and nuts [3]. Food allergies can affect multiple body organs, resulting in a wide variety of clinical manifestations that are usually mild, but may occasionally lead to severe life-threatening reactions. These manifestations include eye symptoms such as tearing; cutaneous symptoms such as pruritus, erythema, urticaria, rash, and angioedema; respiratory symptoms such as sneezing, nasal congestion, cough, dyspnea, and wheezing. Also, gastrointestinal symptoms such as nausea, vomiting, and tongue swelling. If these symptoms are severe enough, anaphylactic shock may occur [3,4].

Similar to food allergy, atopic diseases, namely atopic dermatitis (AD), bronchial asthma, and allergic rhinitis, are another group of diseases in which the underlying pathophysiology involves excess production of either non-IgE or IgE mediated against harmless environmental proteins, such as pollen and dust mite [2,5]. Food allergies have always been linked to atopic diseases, and multiple important associations have been made between them. The coexistence of atopic diseases and food allergies has been noticed and well-documented. One theory explains the genesis of both conditions may be a consequence of abnormal regulation between T helper cells and suppressor T cells, resulting in abnormal production of IgE by plasma cells [6,7]. Nevertheless, as mentioned previously, non-IgE-mediated is also involved in food allergy [2]. In addition, the presence of food allergies can sometimes be used as an indicator of the disease's severity, as it was also well documented that food allergies can exacerbate severe forms of atopic dermatitis, and diet control will provide better control of the disease [8].

Globally, the prevalence of food allergies has varied significantly. For example, in a population-based survey study in the United States, an estimated 10.8% out of more than forty thousand participants were allergic to food [9]. In the United Kingdom, on the other hand, the prevalence of challenge-proven food allergy was reported to be 4% in children less than five years of age [10]. In addition, it was seen that 50% of children with food allergies have atopic dermatitis, while 40% and 30% of children with food allergies have asthma and allergic rhinoconjunctivitis, respectively [5]. Also, clinical studies, in general, have reported that the prevalence of food allergy in AD patients ranges from 20% to 80% [11]. Regionally, a cross-sectional study in Saudi Arabia found the prevalence of atopic diseases to be 27% for bronchial asthma, 13.1% for atopic dermatitis, and 5% for allergic rhinitis [12]. Additionally, another study included asthmatic patients and found that 29% of the sample had clinically documented food allergies [13].

Since food allergies are common in atopic patients, early recognition can positively influence the course of the disease and its manifestations. This study, therefore, aimed to assess the prevalence of atopic diseases among pediatric patients with food allergies in a tertiary care center in Jeddah, Saudi Arabia.

## Materials and methods

Study settings and design

This is a retrospective cohort study that was conducted in King Abdulaziz Medical City (KAMC) in Jeddah, Saudi Arabia.

Identification of study participants

The inclusion criteria for this study were pediatric patients aged between 0-14 years and with a confirmed food allergy within a period from January 2016 to June 2022. 

Data collection process

The data was obtained from the hospital's electronic records. Patients' demographics, such as age, gender, type of food allergy, and symptoms of food allergies, were retrospectively retrieved. Additionally, the presence of atopic diseases, such as atopic dermatitis, asthma, or allergic rhinitis was also documented. Lastly, the number of times of using health care services, including outpatient, inpatient, or emergency room services, was retrieved and reported. The collected data were entered into an Excel file (Microsoft, Redmond, Washington) by the research team and kept confidential. 

Statistical analysis 

The data was checked for completeness and analyzed using JMP Statistical Software version 15.2.0 (SAS Institute, Cary, North Carolina; a subsidiary of the SAS Institute). The data is presented as frequency (%) for categorical variables. The Chi-squared test was utilized for comparison. The significance level was fixed at a p-value of <0.05.

## Results

Demographics and clinical characteristics

A total of 228 patients were identified and included in this study. Of these, 118 (51.8%) were males. The majority of patients' ages were between 5-10 years. The most common food allergens in the study sample were found to be milk products in 88 patients (38.6%), eggs in 81 patients (35.5%), and peanuts in 45 patients (19.7%). The most commonly reported symptoms of food allergy were itching in 128 patients (56.1%), followed by urticaria in 120 patients (52.6%). Among all patients, a total of 114 patients (50%) were diagnosed with an atopic disease. Of these, a total of 74 (64.9%) of them were diagnosed with asthma, and 57 (50%) of them were diagnosed with AD. Allergic rhinitis was the least prevalent with 45 patients (39.5%).

Nearly half of the patients, 93 (41.7%), have had 11-25 outpatient visits in their lifetime, utilizing KAMC health care services (regardless of the reason for their visits), while only 31 patients (13.9%) have visited the hospital as outpatients more than or equal to 51 times. Also, a small proportion of patients, 80 (35.1%), had a history of inpatient admission at least two to four times in their lifetime in KAMC for different reasons. In terms of emergency room (ER) visits, most of our patients, 75 (33.2%), have had five visits or more to ER in KAMC. Specifically, 44 (19.3%) of the included patients visited ER due to a food allergy reaction (Table 1). 

**Table 1:** Demographics and clinical characteristics

Item	N (%)
Age	
< 5 years	81 (35.5%)
5-10 years	111 (48.7%)
11-14 years	36 (15.8%)
Gender	
Male	118 (51.8%)
Female	110 (48.2%)
Food allergy	
Peanut	45 (19.7%)
Milk products	88 (38.6%)
Seafood	28 (12.3%)
Eggs	81 (35.5%)
Meat	5 (2.19%)
Sesame	36 (15.8%)
Strawberry	21 (9.21%)
Banana	27 (11.8%)
Mango	7 (3.07%)
Potato	9 (3.95%)
Chocolates	17 (7.46%)
Pistachio	7 (3.07%)
Soya	6 (2.63%)
Tahina	13 (5.70%)
Nuts	22 (9.65%)
Wheat	33 (14.5%)
Clinical presentation of allergic attack	
Urticaria	120 (52.6%)
Swelling	73 (32%)
Itching	128 (56.1%)
Difficulty swallowing	5 (2.19%)
Wheezing	4 (1.75%)
Dizziness	3 (1.32%)
Nausea	8 (3.51%)
Vomiting	29 (12.7%)
Dyspnea	15 (6.58%)
Abdominal pain	4 (1.75%)
Cough	10 (4.39%)
Angioedema	7 (3.07%)
Atopic disease	
Yes	114 (50%)
No	114 (50%)
Asthma	
Yes	74 (64.9%)
No	40 (35.1%)
Atopic dermatitis	
Yes	57 (50%)
No	57 (50%)
Allergic rhinitis	
Yes	45 (39.5%)
No	69 (60.5%)
Outpatient visits in general	
Less than or equal to 10 visits	43 (19.3%)
11-25 visits	93 (41.7%)
31-50 visits	56 (25.1%)
More than or equal to 51 visits	31 (13.9%)
Inpatients admission in general	
None	47 (20.6%)
1 admission	76 (33.3%)
2-4 admissions	80 (35.1%)
More than or equal to 5 admissions	25 (11%)
Emergency room visits in general	
None	56 (24.8%)
1 ER visit	38 (16.8%)
2-4 ER visits	57 (25.2%)
More than or equal to 5 ER visits	75 (33.2%)
Emergency room visits due to food allergy	
Yes	44 (19.3%)
No	184 (80.7%)

Atopic diseases vs. clinical characteristics

Certain food allergens were associated with the presence of atopy in our patients. A total of 53 (46.49%) patients with atopic diseases have reported an allergy to eggs, and the presence of an egg allergy was significantly associated with the presence of atopy in the patients (p=0.0005). A similar significant association was found between the presence of atopy and the presence of strawberry (p=0.0393), banana (p=0.0242), and chocolate (p=0.0233) allergies. Moreover, in our study, the majority of atopic and non-atopic patients have visited the outpatient clinic 11-25 times in total (p=0.0939). Similarly, the number of total inpatient admissions was similar between atopic and non-atopic patients, with a total of two to four admissions per patient (p=0.5027). Most atopic patients have reported ER visits five times or more in total, while most non-atopic patients have reported no ER visits (p=0.3160). It is important to note, however, that no significant differences were found between atopic and non-atopic patients in the number of times the health care services such as outpatients, inpatients, or ER were used (Table 2).

**Table 2:** Non-atopic disease vs. atopic disease * Significant p-value (<0.05)

Item	Atopic disease, N (%)		p-value
	No	Yes	
Gender			
Male	57 (50%)	61 (53.51%)	0.5960
Female	57 (50%)	53 (46.49%)	
Food Allergy			
Peanut	23 (20.18%)	22 (19.30%)	0.8678
Milk Products	46 (40.35%)	42 (36.84%)	0.5863
Seafood	11 (9.65%)	17 (14.91%)	0.2260
Eggs	28 (24.56%)	53 (46.49%)	0.0005*
Meat	2 (1.75%)	3 (2.63%)	0.6511
Sesame	13 (11.40%)	23 (20.18%)	0.0693
Strawberry	6 (5.26%)	15 (13.16%)	0.0393*
Banana	8 (7.02%)	19 (16.67%)	0.0242*
Mango	1 (0.88%)	6 (5.26%)	0.0549
Potato	3 (2.63%)	6 (5.26%)	0.3076
Chocolates	4 (3.51%)	13 (11.40%)	0.0233*
Pistachio	4 (3.51%)	3 (2.63%)	0.7010
Soya	2 (1.75%)	4 (3.51%)	0.4080
Tahina	6 (5.26%)	7 (6.14%)	0.7752
Nuts	11 (9.65%)	11 (9.65%)	1.0000
Wheat	12 (10.53%)	21 (18.42%)	0.0902
Outpatient visits in general			
Less than or equal to 10 visits	29 (25.44%)	14 (12.84%)	0.0939
11 – 25 visits	45 (39.47%)	48 (44.04%)	
31 – 50 visits	24 (21.05%)	32 (29.36%)	
More than or equal to 51 visits	16 (14.04)	15 (13.76%)	
Inpatients Admission in general			
None	22 (19.3%)	25 (21.93%)	0.5027
1 admission	38 (33.33%)	38 (33.33%)	
2-4 admissions	38 (33.33%)	42 (36.84%)	
More than or equal to 5 admissions	16 (14.04%)	9 (7.89%)	
Emergency room visits in general			
None	32 (28.57%)	24 (21.05%)	0.3160
1 ER visit	19 (16.96%)	19 (16.67%)	
2-4 ER visits	30 (26.79%)	27 (23.68%)	
More than or equal to 5 ER visits	31 (27.68%)	44 (38.6%)	
Emergency room visits due to food allergy			
Yes	22 (19.30%)	22 (19.30%)	1.0000
No	92 (80.70%)	92 (80.70%)	

Most asthma patients in our study have had a concomitant food allergy toward eggs in 39 patients (52.70%), while milk product allergy was documented by 24 patients (32.43%). Similarly, most allergic rhinitis patients had an allergy to eggs (22 patients,48.89%). Eggs and milk product allergies were also the most common food allergens among atopic dermatitis patients in 30 patients (52.63%) and 25 patients (43.86%), respectively. Even though sensitization to certain food allergens has been described variously with atopic diseases, no significant differences were noticed between food allergens and specific types of atopic diseases. Additionally, allergic rhinitis and atopic dermatitis patients have utilized outpatient services 11-25 times in their lifetime, while most asthma patients have utilized it 31-50 times. Also, in terms of inpatient admissions, the majority of asthma patients have been previously admitted two to four times in their lifetime, while most allergic rhinitis and atopic dermatitis patients have only been admitted once previously. It was also noticed that most asthma and atopic dermatitis patients have had a total of five visits or more to the ER in their lifetime. Allergic rhinitis patients, however, had a total of two to four ER visits in their lifetime (Table 3).

**Table 3:** Atopic disease clinical characteristics

	Asthma, N (%)	p-value	Allergic rhinitis, N (%)	p-value	Atopic dermatitis, N (%)	p-value
Food allergen						
Milk products	24 (32.43%)	0.1843	14 (31.11%)	0.3056	25 (43.86%)	0.1204
Eggs	39 (52.70%)	0.0705	22 (48.89%)	0.6785	30 (52.63%)	0.1887
Peanuts	16 (21.62%)	0.3926	11 (24.44%)	0.2608	14 (24.56%)	0.1545
Sesame	16 (21.62%)	0.6007	13 (28.89%)	0.0612	13 (22.81%)	0.4838
Nuts	10 (13.51%)	0.0573	6 (13.33%)	0.2820	4 (7.02%)	0.3413
Seafood	12 (16.22%)	0.5950	7 (15.56%)	0.8763	10 (17.54%)	0.4302
Banana	14 (18.92%)	0.3801	8 (17.78%)	0.7971	13 (22.81%)	0.0785
Strawberry	13 (17.57%)	0.0582	8 (17.78%)	0.2386	6 (10.53%)	0.4059
Tahina	5 (6.76%)	0.7092	4 (8.89%)	0.3236	4 (57.14%)	0.6964
Soya	4 (5.41%)	0.1344	2 (4.44%)	0.661	2 (3.51%)	1
Outpatient visits in general						
Less than or equal to 10 visits	8 (11.27%)	0.1251	5 (11.36%)	0.5951	7 (12.96%)	0.2108
11 - 25 visits	27 (38.03%)		22 (50%)		28 (51.85%)	
31 - 50 visits	26 (36.62%)		13 (29.55%)		11 (20.37%)	
More than or equal to 51 visits	10 (14.08%)		4 (9.09%)		8 (14.81%)	
Inpatients admission in general						
None	17 (22.97%)	0.0885	10 (22.22%)	0.8413	8 (14.04%)	0.1625
1 admission	21 (28.38%)		17 (37.78%)		23 (40.35%)	
2-4 admissions	27 (36.49%)		15 (33.33%)		22 (38.60%)	
More than or equal to 5 admissions	9 (12.16%)		3 (6.67%)		4 (7.02%)	
Emergency room visits in general						
None	13 (17.57%)	0.2143	7 (15.56%)	0.0848	12 (21.05%)	0.9239
1 ER visit	10 (13.51%)		8 (17.78%)		9 (15.79%)	
2-4 ER visits	18 (24.32%)		16 (35.56%)		15 (26.32%)	
More than or equal to 5 ER visits	33 (44.59%)		14 (31.11%)		21 (36.84%)	

## Discussion

Our study aimed to assess the prevalence of atopic diseases among pediatric patients with food allergies and to investigate different subtypes of food allergens in relation to atopic diseases. We also evaluated the volume of utilization of outpatient departments, inpatient admissions, and emergency rooms among these patients.

Our study found that eggs, strawberries, mangoes, and chocolate were more likely to be associated with atopic coexistence in patients with food allergies. Eggs and milk products were the most common allergens in our study, with eggs significantly correlating to the existence of atopic diseases. Another study by Peroni et al. found that skin prick test (SPT) sensitization to eggs was strongly associated with AD. In contrast, milk product allergies were the most prevalent in patients with AD [14].

A recent cross-sectional study conducted in Saudi Arabia showed that allergy to eggs was the most common type of food allergy in all children (30.5%), followed by peanuts (17.1%). In our study, however, milk product allergy was the most common (38.6%), followed by (35.5%) of food allergies due to eggs and (19.7%) due to peanuts. Furthermore, the Saudi Arabian study found that AD was the most common atopic disease to coexist with food allergies, while asthma was the most common in our findings [15].

Several studies have suggested that having a symptomatic allergy to any food allergen is strongly associated with an asthma diagnosis in children and that asthmatic children with food allergies are at increased risk for asthma morbidity and severe asthma [16,17]. In our study, asthma was the less relevant atopic disease with inpatient admissions compared to AD and allergic rhinitis. For ER visits, asthma showed the highest number of patients with at least one visit, followed by AD. A review article by Sarinho et al. showed that the association between food allergies and asthma increases the severity of food allergy reactions and management difficulties [17]. In addition, Emons et al. concluded that coexisting asthma in food allergies had more severe forms of food allergy that could be fatal [16].

The current study must be considered within the context of certain limitations. As this study is single-centered, the sample size was small to draw multiple conclusions as subgroup analysis introduces a small number of subjects per group. As such, we recommend further multi-centered studies with a larger sample size to adequately assess atopic diseases and their associations in food-allergic patients. Also, this study recommends early identification and recognition of those patients to provide the required services and increase their quality of life.

## Conclusions

In conclusion, the current study demonstrates the prevalence of atopic disease in patients with food allergies. Eggs and milk are the more prevalent allergens in patients with atopic diseases in this study. There was a significant association between allergy to eggs, bananas, and strawberries and the presence of atopic diseases. No significant difference was found between atopic and non-atopic patients in terms of outpatients, inpatients, or ER utilizations. As atopic diseases are comorbid conditions in patients with food allergies and could negatively impact the disease course, an early and comprehensive assessment is required in those patients.
